# Seed mass, hardness, and phylogeny explain the potential for endozoochory by granivorous waterbirds

**DOI:** 10.1002/ece3.5997

**Published:** 2020-01-15

**Authors:** Ádám Lovas‐Kiss, Orsolya Vincze, Erik Kleyheeg, Gábor Sramkó, Levente Laczkó, Réka Fekete, Attila Molnár V., Andy J. Green

**Affiliations:** ^1^ Wetland Ecology Research Group Department of Tisza Research MTA Centre for Ecological Research‐DRI Debrecen Hungary; ^2^ Evolutionary Ecology Group Hungarian, Department of Biology and Ecology Babeş‐Bolyai University Cluj Napoca Romania; ^3^ Sovon Dutch Centre for Field Ornithology Nijmegen The Netherlands; ^4^ MTA‐DE ‘Lendület’ Evolutionary Phylogenomics Research Group Debrecen Hungary; ^5^ Department of Botany University of Debrecen Debrecen Hungary; ^6^ Department of Wetland Ecology Estación Biológica de Doñana EBD‐CSIC Sevilla Spain

**Keywords:** *Anas platyrhynchos*, endozoochory, phylogeny, retention time, seed dispersal, seed traits

## Abstract

Field studies have shown that waterbirds, especially members of the Anatidae family, are major vectors of dispersal by endozoochory for a broad range of plants lacking a fleshy fruit, yet whose propagules can survive gut passage. Widely adopted dispersal syndromes ignore this dispersal mechanism, and we currently have little understanding of what traits determine the potential of angiosperms for endozoochory by waterbirds. Results from previous experimental studies have been inconsistent as to how seed traits affect seed survival and retention time in the gut and have failed to control for the influence of plant phylogeny. Using 13 angiosperm species from aquatic and terrestrial habitats representing nine families, we examined the effects of seed size, shape, and hardness on the proportion of seeds surviving gut passage through mallards (*Anas platyrhynchos*) and their retention time within the gut. We compiled a molecular phylogeny for these species and controlled for the nonindependence of taxa due to common descent in our analyses. Intact seeds from all 13 species were egested, but seed survival was strongly determined by phylogeny and by partial effects of seed mass and hardness (wet load): species with seeds harder than expected from their size, and smaller than expected from their loading, had greater survival. Once phylogeny was controlled for, a positive partial effect of seed roundness on seed survival was also revealed. Species with seeds harder than expected from their size had a longer mean retention time, a result retained after controlling for phylogeny. Our study is the first to demonstrate that seed shape and phylogeny are important predictors of seed survival in the avian gut. Our results demonstrate that the importance of controlling simultaneously for multiple traits and relating single traits (e.g., seed size) alone to seed survival or retention time is not a reliable way to detect important patterns, especially when phylogenetic effects are ignored.

## Graphical abstract

Based on a feeding experiment with a granivorous waterfowl, we analyze the variation in endozoochory potential among thirteen vascular plant species. Seed traits (especially seed hardness and seed size) had significant partial effects on the ability of seeds to pass the digestive tract of birds intact, as well as their retention time. Moreover, there was a strong phylogenetic signal in the seed survival of different plant species.

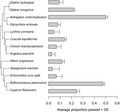

## 1 INTRODUCTION

Migratory waterbirds such as ducks, shorebirds, and gulls disperse a broad range of angiosperms and other plants with propagules capable of surviving passage through the avian gut (i.e., through “endozoochory”, Green et al. 2016; Lovas‐Kiss, Sanchez, et al., 2018b; Lovas‐Kiss, Vizi, Vincze, Molnár, & Green, 2018a; Lovas‐Kiss et al., 2019; Reynolds & Cumming 2016a). Recent models confirm that these birds are major vectors for long‐distance dispersal (LDD), and angiosperm seeds are often moved over hundreds of km within the digestive tract of waterbirds such as granivorous dabbling ducks *Anas* spp. (Farmer, Webb, Pierce, & Bradley, 2017; Kleyheeg et al., 2019; Reynolds & Cumming, 2016b; Viana, Santamaría, Michot, & Figuerola, 2013). Waterbird endozoochory has now been demonstrated for a variety of terrestrial and aquatic plants that lack a fleshy fruit, although this does not coincide with the dispersal syndromes often assigned based on seed morphology (Bartel, Sheppard, Lovas‐Kiss, & Green, 2018; Lovas‐Kiss, Sanchez, et al., 2018b; Lovas‐Kiss et al., 2019; Lovas‐Kiss, Vizi, et al., 2018a). This is a form of “nonclassical endozoochory,” as it does not coincide with the “endozoochory syndrome” (Green, Elmberg, & Lovas‐Kiss, 2019). Diet studies for dabbling ducks suggest they disperse over 500 plant species in Europe alone (Lovas‐Kiss, Vizi, et al., 2018a; Soons, Brochet, Kleyheeg, & Green, 2016). The maximum plant dispersal distances provided by migratory waterbirds greatly exceed those expected from abiotic vectors, and therefore, have an important influence on changes in plant distribution in response to climate change, or biological invasions (Bullock et al., 2017; Viana, 2017).

For a given plant species, the chances of “effective dispersal” (i.e., the production of adult plants, Schupp, Jordano, & Gómez, 2017) by waterbirds depends partly on the ability of its seeds to resist digestive processes and to be egested from the digestive tract intact, as well as the retention time within the gut (Green et al. 2016; Kleyheeg, Nolet, Otero‐Ojea, & Soons, 2018b). The proportion of seeds surviving gut passage (i.e., seed survival) and retention time depends partly on variation in the diet and gut morphology of the vectors, as well as the amount and type of grit in the gizzard (Figuerola & Green, 2002; Kleyheeg, Nolet, et al., 2018b). On the other hand, seed traits can be expected to have a major influence on both seed survival and retention time, although the relative importance of different traits for plants lacking a fleshy fruit remains unclear. There is evidence to suggest that seed size *per se* is important, as would be expected since very large seeds may be unable to pass from the gizzard into the intestines (Soons et al., 2016). In an experiment with mallards and 23 wetland plant species, Soons, Vlugt, Lith, Heil, and Klaassen (2008) found seed survival of up to 54%, with smaller seeds having significantly greater survival and faster passage. Other experimental studies found no relationship between seed size and survival during passage through the guts of dabbling ducks (Brochet, Guillemain, Gauthier‐Clerc, Fritz, & Green, 2010; Wongsriphuek, Dugger, & Bartuszevige, 2008), although a meta‐analysis did support such a size effect (van Leeuwen, Velde, Groenendael, & Klaassen, 2012).

The main reason for the lack of consistency in seed size effects among studies is that other seed traits are likely to be important and that they may covary with size. De Vlaming and Proctor (1968) suggested that small but hard seeds have the highest seed survival. Wongsriphuek et al. (2008) found that seed survival through mallards increased with the fiber content of the seeds. García‐Álvarez et al. (2015) compared four plant species and found that a species with a large seed (*Ludwigia grandiflora*) had the highest seed survival and suggested that this was because it was harder and rounder than the other seeds. Rounder seeds were more likely to germinate from ungulate dung (Albert, Mårell, Picard, & Baltzinger, 2015a; Pakeman, Digneffe, & Small, 2002), and although the importance of seed shape has not previously been tested in waterbirds, we would expect round seeds (e.g., *Cuscuta*, Costea et al., 2016) to have a greater seed survival because they are likely to be mechanically more resistant to crushing in the gizzard. Seeds that are more permeable to water may also be more easily digested by waterbirds (Kleyheeg, Claessens, & Soons, 2018a).

To date, most studies of waterbird endozoochory recognize that different traits can affect seed survival and retention time but without considering how traits covary, or quantifying the partial effect of one trait while controlling for others. For example, seed hardness is positively correlated with seed size (Kleyheeg, Claessens, et al., 2018a). Another limitation of previous studies is that they have not assessed the importance of seed shape. Furthermore, none of these previous studies has attempted to control for phylogenetic effects. De Vlaming and Proctor (1968) suggested that phylogeny is important, with Cyperaceae being better at surviving gut passage through waterbirds than Poaceae or Asteraceae. Because species are related to each other through common descent, they should not be regarded as independent data points in a given study, as they are likely to be more similar in other important and influential characteristics not considered in that study (Grafen, 1989). Closely related species have similar traits, and when studies relating seed traits to the survival of gut passage have not controlled for phylogeny, it is unclear whether phylogenetic relatedness is driving the patterns attributed to a particular trait, or whether there is a causal relationship with the trait *per se*. Controlling for phylogeny thus allows us to control for possible confounding effects. Hence, some studies of the influence of seed traits on endozoochory by ungulates and fish have controlled for phylogeny (Boedeltje et al., 2015; D'hondt & Hoffmann 2011).

In this study, we assess the role of seed traits on their survival and retention time during passage through the mallard digestive tract. Mallards are one of the most numerous and widely distributed waterbird species of the Holarctic region and are good vectors for LDD (Farmer et al., 2017; Kleyheeg et al., 2019; Soons et al., 2016). We aimed to tease apart the relative importance of seed size, hardness, water permeability, and shape, while controlling for the effect of phylogenetic relatedness. We hypothesized that multiple traits are important and that seed size and permeability would have negative partial effects on both survival and mean retention time, while hardness and roundness would have positive partial effects. We also hypothesized that phylogeny has a significant effect on model estimates and results from models excluding phylogeny would be different, and that results with traits explaining less variation.

## 2 MATERIALS AND METHODS

In order to investigate the influence of seed traits and phylogeny on endozoochory potential, we conducted a controlled experiment in captivity. We force‐fed captive‐bred mallards with a mixture of known numbers of seeds of 13 angiosperm species that are lacking fleshy fruits (Table 1, Table S1). All seeds were collected during late summer from different habitats in Hungary. These plants cover 9 plant families, including aquatic and moist soil species and one dry forest taxon (*Lychnis coronaria*). We included Pannon‐basin endemics (*Cirsium brachycephalum*), as well as rare (*Angelica palustris*) and common (*Sparganium erectum*) European species. They ranged from very small (*Cyperus flavescens*, seed volume: 0.016 mm^3^) to large‐seeded species (*Sparganium erectum*, seed volume: 202 mm^3^, Table S1). Only three of the species have previously been used in experiments on endozoochory by waterbirds (*Astragalus contortuplicatus*, *Echinochloa crus‐galli* and *Sparganium erectum* were used by Brochet et al., 2010; Kleyheeg, Nolet, et al., 2018b; Lovas‐Kiss et al., 2015; Mueller & Van der Valk, 2002; Wongsriphuek et al., 2008). For those taxa with capsules and pods, we first removed the seeds from the dry fruits to be able to count them before feeding them to ducks.

**Table 1:** (a) Numbers of events when seeds were fed to ducks (three trials with up to eight ducks) then subsequently recovered intact, in relation to the total number of such events and the total number of seeds ingested during the experiments. (b) Number of intact seeds retrieved, and the median, mean, maximum, and mode of retention time

Plant species	(a)		(b) Retention time^a^				
	Retrieval events/Ingestion events	Seeds ingested	*n*	Med	Mean	Max	Mode
*Allium angulosum*	21/24	2,194	257	4	6.07	31	4
*Angelica palustris*	13/24	2,095	32	4	8.09	21	4
*Astragalus contortuplicatus*	24/24	2,298	1,173	4	5.94	45	4
*Bolboschoenus planiculmis*	16/16	1,555	758	4	8.26	45	4
*Cirsium brachycephalum*	15/16	1,505	95	4	5.67	31	4
*Cuscuta lupuliformis*	24/24	2,167	312	4	5.6	45	4
*Cyperus flavescens*	24/24	2,350	577	4	4.64	21	4
*Echinochloa crus‐galli*	19/24	2,222	150	4	5.63	31	4
*Elatine hungarica*	8/8	795	173	4	4.17	31	4
*Elatine hydropiper*	13/16	1,598	151	4	4.04	7	4
*Glycyrrhiza echinata*	19/24	1,037	82	4	6.09	31	4
*Lychnis coronaria*	16/24	2,253	100	4	5.74	31	4
*Sparganium erectum*	1/24	802	3	4	5	7	4

a These times are a proxy for retention time, and true retention times would be lower at some unknown point between the intervals when feces were collected. For example, a maximum of 45 hr refers to a true maximum somewhere between 31 and 45 hr.

We conducted three consecutive feeding trials, during 22–24 October, 3–5 November, and 17–19 November, respectively. During each trial, each of the eight individual mallards was force‐fed with 100 seeds of the small‐seeded taxa and 50 seeds of the two larger‐seeded species (*Glycyrrhiza echinata, Sparganium erectum*). Prior to the experiments, the seeds were stored dry in paper bags in the refrigerator at 4°C. Three trials were used to increase statistical power and to obtain more reliable results. The species composition of seeds ingested varied slightly among the three trials due to limited availability of certain seeds. Nine species were fed in every trial, while owing to shortage of seeds *Elatine hungarica* was only used in the first, *Cirsium brachycephalum* only in the first and second, and *Elatine hydropiper* and *Bolboschoenus planiculmis* only in the second and third trials. Prior to the experiments, and in between feeding trials, mallards were housed communally in outdoor facilities and fed with mixed grains (corn, wheat, oat) and green leaves (e.g., *Stellaria media, Taraxacum officinale*). Grit was freely available to the birds outside the experimental trials. Twenty‐four hours prior to force‐feeding, ducks were moved to individual cages (50 × 50 × 50 cm) where no food was provided, to ensure their digestive tracts were relatively empty and to minimize potentially confounding effects of other food items in the digestive tract. Water was provided ad libitum throughout the study. Individual cages were built of wire mesh and a clean plastic sheet was placed under each cage once force‐feeding was completed, to facilitate fecal sample collection.

Force‐feeding was done using a small plastic cone, placed in the bird's throat. All seeds were gently poured into the esophagus while ensuring that every seed was ingested. Although this feeding method bypasses seed handling within the bill by the birds, this is not expected to influence seed survival since ducks do not damage seeds within their soft bill (Gurd, 2007). Following force‐feeding, some mallards regurgitated a small proportion of seeds, which were counted and subtracted from the number of ingested seeds before statistical analyses. Droppings were collected from the sheets placed under cages at five intervals following force‐feeding, at 4, 7, 21, 31, and 45 hr postfeeding. After 45 hr, the trials were ended, and the ducks returned to a communal pen. Hence, we assumed that all seeds that survived gut passage were egested within 45 hr, as suggested by the small proportion of seeds recovered after 31 hr or later. Fecal samples were left to dry at room temperature, then the intact seeds in each sample were collected and counted under a binocular microscope. We considered seeds to be intact if there were no visible cracks or damage to their coat. Germination tests were then run for 195 days, and results were compared to those of control seeds that had not been fed to ducks (see Costea et al.^2019^ for details).

Mallards were obtained from a local breeder and were 1 year old at the time of the experiment. They showed no signs of ill effects after the experiment and were returned to the local breeder. The experiment was approved by the scientific council of the Babeş‐Bolyai University of Cluj Napoca (reference number: 14689/31.08.2018).

### 2.1 Seed traits

In order to investigate how seed traits influenced seed survival and retention time, we measured the following five traits for seeds that had not been ingested by birds. Each character was measured on 50 seeds (10 in the case of hardness and shape), and these values were averaged for the species (Table S1).

#### 2.1.1 Hardness

To measure hardness, we used an Instron 5542 machine (Kleyheeg, Claessens, et al., 2018a). The seeds were placed under a metal pin that was slowly lowered by the machine (0.1 mm/s) while measuring the force required to lower the pin (measurements taken every 0.1‐s interval). Once the pin reaches the seed, pressure builds up until the seed cracks and the pressure drops briefly. “Cracking load” was therefore defined as the maximum load (in kg) before the pressure dropped. We measured both dry and wet seed hardness (dry and wet load) for each species. Seeds were soaked in distilled water for 2 hr prior to measurements of wet load. Most measurements were based on 10 randomly selected seeds per species. However, occasionally no clear pressure peak could be identified, and these seeds were excluded from the tests and replaced.

#### 2.1.2 Thousand seed mass

For each plant species, we measured the mass of a batch of 100 dry seeds with an analytical balance (precision 0.0001 g). The measurement was repeated with three batches of seeds, and values were averaged for each species. To obtain thousand seed mass (the standard measure in plant trait databases), we multiplied the values obtained by 10 (Lovas‐Kiss et al., 2015).

#### 2.1.3 Volume

High‐resolution macro photos using a stereomicroscope were taken for 50 seeds of each species from two different angles using millimeter paper as a scale, so that three dimensions (height, width, and depth) could be measured from the photos. Seed measurements were taken with a digital caliper (precision 0.01 mm) from the photographs in the software *tpsDig2* (version 2.29.). Seed volume was estimated as the product of the three dimensions. We used the average for the 50 seeds measured.

#### 2.1.4 Water permeability

The above method was used to estimate the volume of 10 seeds of each species when dry. Each seed was then placed in a clean Eppendorf tube and submerged in distilled water for 2 hr at room temperature. Wet seeds were then immediately photographed a second time to estimate their volume again, using the same axes as when dry. Water permeability was then considered to be the ratio between wet seed volume and dry seed volume.

#### 2.1.5 Shape

For the quantification of seed shape, we used the means of measurements of seed length, width, and height of 10 seeds, applying the formula described by Bekker et al. (1998) to calculate a shape index that varies from 0 (perfect sphere) to 0.2 (slim disk or a thin needle). Earlier studies on endozoochory (Albert, Auffret, et al., 2015b; Albert, Mårell, et al., 2015a) have also used the Bekker shape index to describe seed shape.

#### 2.1.6 Phylogeny

**Figure 1:**
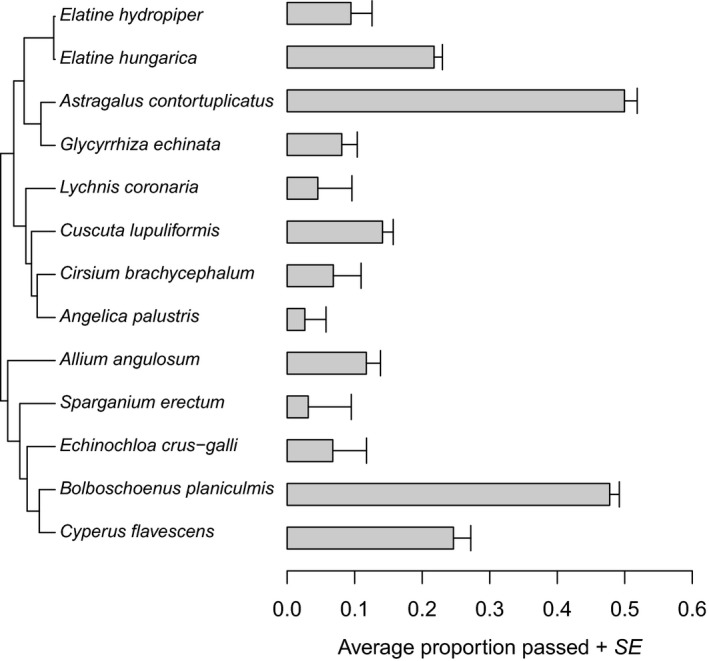
Proportion of seeds surviving gut passage for different plant species (mean ± *SE* for different ducks/trials), plotted alongside the molecular phylogeny. Accession numbers of sequences obtained from GenBank: *Allium angulosum*: LN867002; *Bolboschoenus planiculmis*: GQ130341; *Cuscuta lupuliformis*: DQ924570; *Cyperus flavescens*: KF150598; *Echinochloa crus‐galli*: AB353387; *Elatine hungarica*: KX555590; *Elatine hydropiper*: KX555592; *Glycyrrhiza echinata*: U56000; U55999; *Lychnis coronaria*: AY857966; *Sparganium erectum*: KF265394

For 10 of the 13 species used in this study, nrITS sequences were available from GenBank (Clark, Karsch‐Mizrachi, Lipman, Ostell, & Sayers, 2016). For the remaining three species (*Astragalus contortuplicatus*, *Cirsium brachycephalum*, and *Angelica palustris*), we amplified and sequenced the nrITS region. Field tissue samples were stored in silica gel prior to extraction (Chase & Hills, 1991). A modified CTAB extraction protocol was used to obtain DNA extracts, as detailed in Sramkó et al. (2016). Universal primer ITS4 (White, Bruns, Lee, & Taylor, 1990) and angiosperm‐specific primer ITS1A described by Gulyás et al.. (2005) were used to amplify the target DNA region using the PCR conditions as described in Sramkó et al. (2016). Specific amplicons were submitted to a commercial sequencing service provided by Macrogen Inc. (Korea). Obtained sequences were aligned using the Muscle algorithm as implemented in BioEdit v.7.1.3 (Hall, 1999). Phylogenetic relationships of the species were reconstructed in Paup v.4.0b*10 (Swofford, 2003) by using a heuristic search that was constrained by a guide tree that described the phylogenetic relationships of the major clades of Angiosperms. Therefore, the phylogenetic reconstruction was used to specify the phylogenetic distance among the studied species (i.e., to quantify evolutionary relatedness between them). The robustness of the obtained tree was tested with nonparametric bootstrap using 1,000 pseudoreplicates. The single most parsimonious phylogram was converted into an ultrametric tree by r8s (Sanderson, 2003). This ultrametric tree (Figure 1) was used in all downstream analyses using phylogenetic control.

### 2.2 Statistical analysis

#### 2.2.1 Passage dynamics of seeds

To test how the temporal dynamics of seed egestion varied among different plant species, we used linear mixed‐effect models. The proportion of all seeds egested for a given species that was recovered at a given time interval was used as the dependent variable, while the time of sample collection was included as a fixed factor in the model. The random part of the model included trial ID, mallard ID, plant species random intercept terms and a slope term for each plant species at each time of sample collection. The effect of this random slope term indicates how different the temporal dynamics of seed passage are for different plant species and was assessed using likelihood‐ratio statistics, comparing the above‐described model to a similar model with the random slope term removed.

#### 2.2.2 Seed traits and their influence on the proportion of seeds surviving

In order to investigate how seed egestion was related to seed traits for each plant species, we calculated the proportion of all seeds ingested that was recovered from fecal samples, calculated separately for each individual mallard and trial (*n* = 272 observations). We built linear mixed‐effect models with the proportion of seeds recovered as a dependent variable and four seed traits (wet load, seed mass, water permeability ratio, and dry seed shape) included as covariates. We excluded dry seed volume because it was highly correlated with seed mass (Table S2), and the latter was measured with greater precision. Dry and wet load were also highly correlated (Table S2), and we excluded dry load because wet load explained slightly more variation in seed passage rates in our final models. The above analyses were repeated using Bayesian MCMCglmm in the R package (MCMCglmm, Hadfield, 2010) in order to incorporate phylogenetic inertia into the calculations. In this latter model, we used the same dependent and explanatory variables, the same random terms, and a correlation structure built based on the phylogenetic tree (Figure 1). All continuous predictor variables were log‐transformed prior to the analyses to ensure homoscedasticity. We calculated Lynch's phylogenetic heritability, as a measure of phylogenetic signal. We report posterior mean of the heritability as well as the 95% highest posterior density (HPD) interval based on MCMC draws from the posterior distribution.

#### 2.2.3 Influence of seed traits on retention time

For each plant species, mean retention time was calculated for intact, egested seeds (Table S1). The calculation was performed separately for each individual mallard, and for each of the three trials. For some plant species, no seeds were recovered in the feces of certain individual mallards, hence sample size varied among plant species. Retention time was analyzed using linear mixed‐effect models. These included mean retention time as a dependent variable, while seed traits (wet load, seed mass, water permeability ratio, dry seed shape) were included as covariates. Plant species, trial ID, and mallard ID were introduced into the model as random factors. All continuous variables were log‐transformed prior to analyses. The above model was rerun using Bayesian MCMCglmms including phylogenetic relatedness.

## 3 RESULTS

### 3.1 Phylogenetic reconstruction

The heuristic search using the maximum parsimony criterion found a single most parsimonious constrained phylogenetic tree at 1,207 steps (consistency index = 0.7167; homoplasy index = 0.2833; retention index = 0.5804). The resulting phylogenetic tree appeared to be acceptable (i.e., all branches received *b*s >50) and accurately matched the well‐established phylogenetic relationship for Angiosperms (Stevens, onwards).

### 3.2 Seed survival for different plant species

Intact seeds of all thirteen species used were recovered from the feces of mallards (Table 1). There was a tendency for seed survival to decrease between the experimental trials. In the first feeding trial, there was an overall seed survival of 22%, dropping to 17% and 11% in the second and third trials, respectively.

The average proportion of seeds that survived gut passage varied greatly among species (χ^2^ = 198.76, *df* = 1, *p* < .0001, Figure 1., Table 1). Differences among species remained strong when controlling for phylogeny (ΔDIC = 251.07). However, heritability in this model was high (mean = 0.79, 95% HPD interval 0.65–91), indicating a strong phylogenetic effect (i.e., closely related species tended to have similar levels of seed survival, Figure 1.).

The taxa with the highest levels of overall seed survival were *Astragalus contortuplicatus* (51.0%) and *Bolboschoenus planiculmis* (48.7%). *Sparganium erectum* had the lowest survival (0.37%, Table 1). *Elatine hungarica* had more than twice the seed survival than its congener *E. hydropiper* (21.8% compared to 9.4%), but this may largely be because only *E. hungarica* was used in the first trial (see Section 2).

The viability of recovered seeds was confirmed by germination tests for all taxa except *Sparganium erectum*, for which no control seeds germinated either. Similarly, only one control seed and one passed seed germinated for *Angelica palustris*. For the other 11 species, germinability of passed seeds ranged from 3% to 83%, compared to 0% to 78% for control seeds (details in Costea et al.^2019^).

### 3.3 Passage dynamics of seeds of different plant species

**Figure 2:**
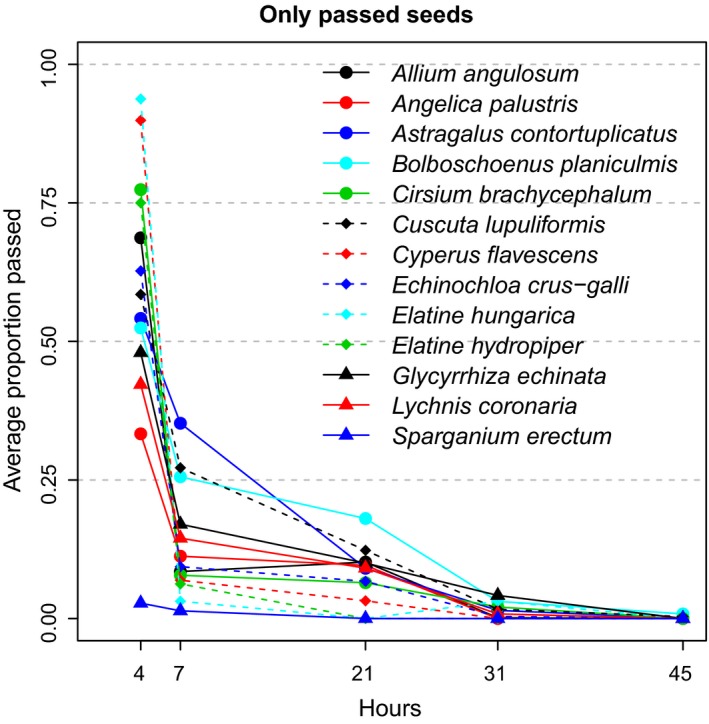
Dynamics of seed passage, showing overall proportions of intact seeds recovered at each time period for each plant species

**Figure 3:**
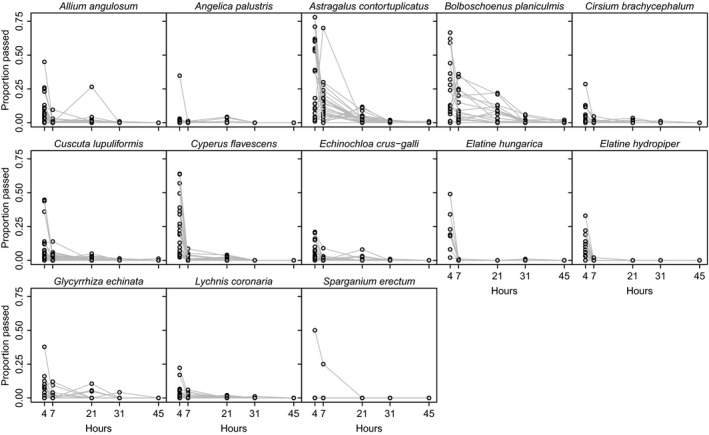
Proportion of ingested seeds recovered intact after 4, 7, 21, 31, and 45 hr for each duck event. Each line represents a different duck event (from three trails, each using eight ducks)

Passage dynamics (i.e., the temporal pattern of seed egestion) differed greatly among plant species (Figures 2 and 3), as indicated by linear mixed models and a significant increase in model fit when seed passage dynamics were estimated separately for each species (χ^2^ = 127.98, *df* = 1, *p* < .0001). Controlling for phylogeny in this model was not possible since correlation structure and interactions could not both be included for the same random effect.

For all plant species, the majority of intact seeds were retrieved within 7 hr of ingestion (Figures 2 and 3). Two taxa (*Elatine hydropiper* and *Sparganium erectum*) had a maximum retention time of 7 hr after ingestion, with the other 11 species having longer maxima (Table 1). *Astralagus contortuplicatus*, *Cuscuta lupuliformis*, and *Bolboschoenus planiculmis* all had a few intact seeds recovered after 45 hr.

### 3.4 Seed traits and their influence on seed survival

**Figure 4:**
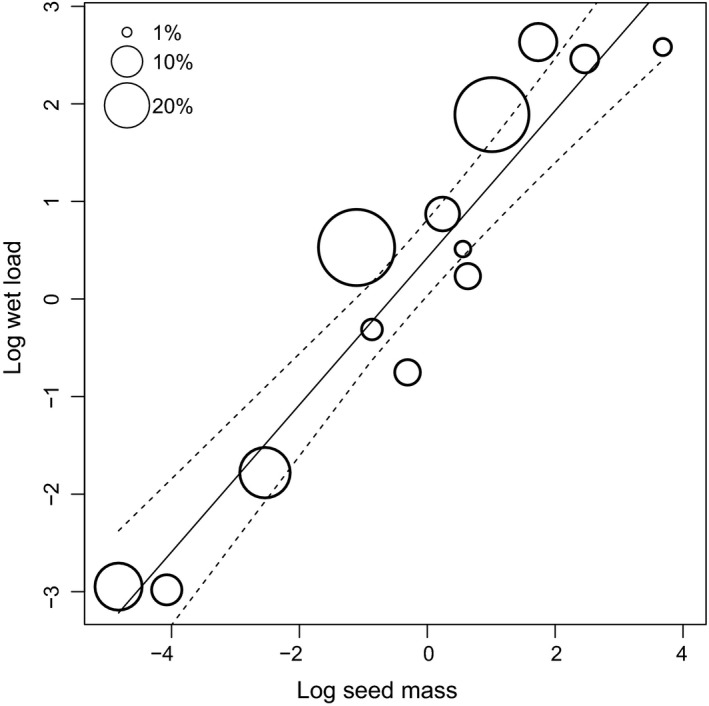
Seed survival after gut passage in relation to thousand seed mass (g) and wet load (kg) for 13 plant species. The size of the circles is proportional to the percentage of ingested seeds that survived gut passage. Slope and associated 95% confidence intervals represent the association between wet load and seed mass, estimated based on a linear regression

Some traits were strongly correlated, especially seed volume and mass (Table S2). Volume was excluded from the models presented (see Section 2). According to partial effects in linear mixed models, seed survival increased in species with greater wet load and with lower seed mass (Table 2a, Figure 4). The direction of these trait associations was similar in univariate models, but the effect of either trait only reached statistical significance once the other trait was controlled for (i.e., only the partial effects of these variables were statistically significant). Hence, seed survival is highest for seeds that are harder than expected based on their size and smaller than expected based on their hardness (Figure 4). There were highly significant differences between individual ducks in the degree of seed survival, and a significant difference between trials (Table 2a).

**Table 2:** (a) Linear mixed‐effects model of the proportion of seeds passed in response to seed traits for 13 plant species, showing their partial effects. The effect of random terms (marked by italics) was assessed by likelihood‐ratio statistics. (b) A similar model run using MCMCglmm and incorporating phylogenetic relatedness

(a)	β (*SE*)	χ2	*df*	*p*
log(wet load)	0.20 (0.06)	9.31	1	.0022
log(seed mass)	−0.16 (0.05)	8.75	1	.0031
log(ratio water permeability)	−0.41 (0.47)	0.74	1	.9623
log(shape dry)	0.15 (0.09)	2.75	1	.0972
*Duck ID*		*21.17*	*1*	*<.0001*
*Trial*		*5.99*	*1*	*.0143*
*Plant species*		*77.94*	*1*	*<.0001*

(b)	post.mean	LCI	UCI	eff.samp	pMCMC
(Intercept)	0.71	−0.07	1.41	990	0.0343
log(wet load)	0.20	0.09	0.29	990	0.0040
log(thousand seed mass)	−0.17	−0.25	−0.08	990	0.0040
log(ratio water permeability)	−0.59	−1.46	0.24	1,150	0.1495
log(shape dry)	−0.18	0.01	0.37	990	0.0383

When using MCMCglmm to control for phylogeny, these effects of wet load and mass remained the same, while the partial effect of shape also became significant (Table 2b). Seed survival was favored by greater roundness. Heritability was high (mean = 0.57, 95%, HPD interval 0.29–0.83), indicating a strong phylogenetic effect (i.e., closely related species tended to have similar levels of seed survival, even after controlling for seed traits).

### 3.5 Influence of seed traits on retention time

**Figure 5:**
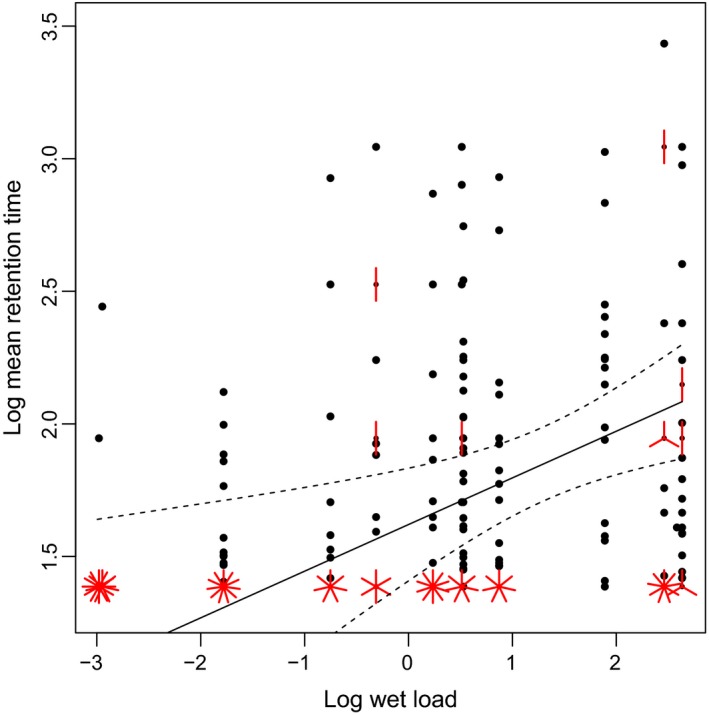
Relationship between seed hardness (wet load) and mean retention time shown on a sunflower plot. Dots represent single data points, while the number of petals shows the number of data points with similar parameter values. Slope and associated 95% confidence intervals were obtained from the model presented in Table 3a

Because some mallard individuals passed no intact seeds of particular plant species (e.g., *Sparganium erectum* seeds were only recovered from one mallard and one trial), we limited the analysis of retention times to a restricted number of individual ducks and plant taxa. According to partial effects in linear mixed models, wet load had a strong partial effect on retention time, with relatively harder seeds being retained longer in the gut (Table 3a, Figure 5). This wet load effect was similar in univariate models. There were also highly significant differences in retention time between individual ducks (Table 3a). When using MCMCglmm to control for phylogeny, the partial effect of wet load was more significant (Table 3b). Heritability in the model was zero (95% conf intervals 0.00–0.00), indicating a very low phylogenetic signal in retention time. The marginally significant effect of seed mass after phylogenetic correction suggests that, for a given wet load, smaller seeds were retained for longer in the alimentary canal (Table 3).

**Table 3:** (a) Linear mixed‐effects model of mean retention time, giving partial effects of seed traits. The effect of random terms (marked in italics) was assessed using likelihood‐ratio statistics. (b) A similar model run using MCMCglmm and including phylogenetic relatedness.

(a)	β (*SE*)	χ2	*df*	*p*
log(wet load)	0.18 (0.06)	7.95	1	.0048
log(thousand seed mass)	−0.07 (0.05)	2.22	1	.1359
log(ratio water permeability)	0.39 (0.44)	0.79	1	.3754
log(shape dry)	0.03 (0.08)	0.13	1	.7139
*Duck ID*		*27.74*	*1*	*<.0001*
*Experiment*		*0*	*1*	*1*
*Plant species*		*0*	*1*	*1*

(b)	post.mean	LCI	UCI	eff.samp	pMCMC
(Intercept)	1.74	1.15	2.25	990	<0.001
log(wet load)	0.18	0.07	0.28	990	<0.001
log(thousand seed mass)	−0.08	−0.17	0.01	990	0.0889
log(ratio water permeability)	0.39	−0.32	1.29	990	0.3111
log(shape dry)	0.03	−0.13	0.18	990	0.6141

## 4 DISCUSSION

Our study provides an important advance in the understanding of how traits of angiosperms lacking a fleshy fruit can determine their dispersal potential, through influencing seed survival and retention time when ingested by avian vectors such as dabbling ducks. We found that seeds of all of the 13 species tested can pass the avian gut intact, with up to 51% overall survival. The viability of recovered seeds was confirmed by germination tests for all taxa except *Sparganium erectum*, although the germinability was not strongly related to traits (Costea et al. 2019 ). Furthermore, there was no significant difference between germinability of recovered seeds and control seeds for 11 of the taxa, whereas passage increased germinability for *Lychnis coronaria* and decreased it for *Elatine hungarica* (Costea et al.^2019^). Hence, in general, germination is not a better indication of seed survival than egestion (see Kleyheeg, Claessens, et al., 2018a for a similar study of germinability in relation to seed traits). Seed survival and mean retention time varied greatly between individual ducks, as reported in previous experimental studies (Green et al. 2016), and also for other seed vectors such as fish (Pollux, 2017).

So far, our understanding of which plant species are dispersed by endozoochory by migratory waterbirds is limited by a general lack of empirical studies. However, those available show that plant species dispersed are associated with a range of terrestrial and wetland habitats and cannot be adequately predicted by simple classifications such as those commonly used to identify putative dispersal syndromes based on visual inspection of seed morphology (Bartel et al., 2018; Lovas‐Kiss et al., 2019; Lovas‐Kiss, Vizi, et al., 2018a; Soons et al., 2016). Indeed, this failure is inevitable since, by definition, no plants lacking a fleshy fruit can be assigned to the “endozoochory syndrome” (Pérez‐Harguindeguy et al., 2013). Hence, studies such as ours are a vital step toward predicting endozoochory potential based on traits (see also Green et al., 2019). Our study only included a limited part of the size spectrum for angiosperm seeds, and field studies suggest that Anatidae are less likely to disperse plant species with larger seeds (Hattermann, Bernhardt‐Römermann, Otte, & Eckstein, 2019; Soons et al., 2016).

Our results suggest that endozoochory by waterfowl may be a viable dispersal mechanism for each of the 13 species. Although to our knowledge, only three of them (*Echinochloa crus‐galli*, *Elatine hydropiper*, *Sparganium erectum*) have been recorded in the alimentary tract of dabbling ducks, congeners of the other species have been recorded in ducks or geese (Costea et al., 2016; Hattermann et al., 2019; Soons et al., 2016). Dispersal of our study species by mallards is likely, since the list of plant species ingested by ducks is far from complete, and there is a lack of diet data from central Europe (Lovas‐Kiss, Vizi, et al., 2018a; Soons et al., 2016). We found here that not only do widespread, common plant species have strong potential for endozoochory, but also rare species with limited distributions, such as *Astragalus contortuplicatus*, the glacial relict *Angelica palustris*, or the Pannon‐basin endemic *Cirsium brachycephalum*. This indicates that, although the potential to undergo LDD through waterbirds is a widespread phenomenon among angiosperms, other factors such as environmental filtering play an important role and may prevent effective dispersal, limiting the ultimate distribution patterns of plant species (Fraaije, Braak, Verduyn, Verhoeven, & Soons, 2015; Lovas‐Kiss et al., 2015). Future field studies should attempt to confirm that dabbling ducks feed on range‐restricted species, such as *Cirsium brachycephalum* and *Angelica palustris*, to obtain a better understanding of the relative importance of dispersal limitation versus environmental filtering in the spatial dynamics of such plant species.

In this experimental study, we addressed the influence of multiple seed traits on gut passage. Using a new set of species (10 of our plant taxa were never used in earlier experimental studies of waterbird endozoochory), our results supported those previous studies which suggested that both the size of seeds and measures of their hardness (Brochet et al., 2010; Kleyheeg, Claessens, et al., 2018a; Reynolds & Cumming, 2016b) can influence seed survival during passage through the gut of waterbirds, as well as retention time. However, other studies found contradictory results (see introduction), and this can be explained by the frequent tendency to relate seed survival and retention time to individual seed traits, one at a time.

Different traits used in studies of seed survival during endozoochory are interdependent and correlated (Table S2). In particular, many of them are related to “hardness” and, other things being equal, we can expect load (a measure of hardness or structural strength) to be positively correlated with roundness and negatively correlated with water permeability. We can also expect load to be positively correlated with traits used in other studies such as seed coat thickness (Soons et al., 2008) or fiber contact (Wongsriphuek et al., 2008). In our study, the partial effects of seed mass and wet load were similar for seed survival and retention time, although mass was only statistically significant for seed survival. This indicates that seeds that are stronger than expected from their size are both more likely to survive gut passage, and likely to be retained longer before egestion, and hence be dispersed over greater distances. The strong partial positive effect of wet seed load was not surprising. Kleyheeg, Nolet, et al. (2018b) showed experimentally that this is explained by the capacity of harder seeds to survive for longer in the gizzard, where mechanical digestion takes place before seeds are released into the intestines. Harder seeds are therefore more likely to survive gut passage and are egested over a longer span of retention times than soft seeds, with greater mean and maximum retention times, increasing the chances of LDD events and the maximum dispersal distance (Farmer et al., 2017; Kleyheeg et al., 2019).

When relating traits one by one to seed survival, the results are unpredictable and may be misleading, especially if there is no attempt to control for phylogeny. For example, if only seed size is considered and related to seed survival, we can expect the results to differ between a set of species where small seeds are relatively harder than larger ones, and a second set where small seeds are relatively softer than larger ones. Only by looking at partial effects of size and hardness can we expect to find consistent results. In a study relatively similar to ours, Reynolds and Cumming (2016b) used dry load as a measure of seed hardness for seven different plant species (none of which were included in our study) fed to two African Anatidae and found mass and load to be positively correlated. They analyzed the partial effects of seed length and load on seed survival and retention time and obtained similar results to us. Load had a positive partial effect and length a negative partial effect on retention time. Length had a negative partial effect on seed survival, whereas the effect of load was not significant (Reynolds & Cumming, 2016b). Many other studies did not analyze partial effects but instead only carried out simple correlations and were not able to adequately tease apart the role of different traits (see Introduction).

Our analysis controlling for phylogeny revealed a significant effect of shape on intact gut passage, providing evidence that rounder seeds are more likely dispersed by waterfowl than elongated seeds, similar to earlier findings in ungulates (Albert, Mårell, et al., 2015a; Pakeman et al., 2002). Again, we only detected the role of seed shape because we looked at partial effects while controlling for the more important traits of load and size. Our results suggest that there is much in common between endozoochory by waterbirds and by ungulates. Indeed, there is considerable overlap in the angiosperm taxa dispersed by these two kinds of vectors (Lovas‐Kiss et al., 2019; see also the overlap between the lists of Albert, Mårell, et al., 2015a and Soons et al., 2016).

Our study confirms for the first time that the phylogeny of the plants used in an experiment has an important effect on seed survival during avian gut passage and suggests that failure to control for phylogeny in all previous studies of waterfowl endozoochory may have influenced their results. The phylogenetic signal for seed survival was probably underestimated because the congeneric *Elatine* species were used in different feeding trials, which was likely to overestimate their differences in seed survival. The effect of seed shape was only revealed when we controlled for phylogeny. An effect of phylogeny means that closely related taxa respond more similarly to a treatment (e.g., mallard digestion) than unrelated species. This is because related species are more likely to share morphological or structural traits relevant to their survival, but which are not specifically controlled for in the analysis. Such unmeasured traits might include, for example, physiological processes, or location and structural nature of different tissues, that are shared between related taxa and affect seed survival. Vazačová and Münzbergová (2014) also showed that controlling for phylogeny is crucial to detect relationships between plant seed traits and their island distributions.

The present study demonstrates the effect of multiple seed traits on passage through the digestive tract of dabbling ducks, and therefore, their dispersal potential. This study is the first to show that seed shape and phylogeny have an important influence on avian endozoochory. Broader studies with more plant and bird species are needed to further improve our understanding of seed traits that are important for dispersal by waterfowl and other vectors of nonclassical endozoochory such as shorebirds, gulls, or corvids (Green et al., 2019; Lovas‐Kiss, Sanchez, et al., 2018b; Lovas‐Kiss et al., 2019). Controlling for phylogeny in future experimental feeding studies is recommended to help interpret the effects of traits of interest. In the future, this will allow us to improve predictions of LDD events, by allowing models to take into account key seed traits when predicting retention times and survival (Kleyheeg et al., 2019; Viana et al., 2013). In turn, this may help us improve predictions of the response of plant populations to climate change (Kleyheeg et al., 2019; Viana, 2017), or the spread of alien species (Green, 2016).

## CONFLICT OF INTEREST

None declared.

## AUTHORS’ CONTRIBUTIONS

ÁL‐K, AMV, AJG conceived the ideas and designed methodology; ÁL‐K, RF, LL, EK, SG collected the data; OV, ÁL‐K analyzed the data; ÁL‐K, OV, AJG, EK led the writing of the original manuscript, and ÁL‐K and AJG the revisions. All authors contributed critically to the drafts and gave final approval for publication.

## Data Availability

All data used in the analyses will be available from Dryad—https://doi.org/10.5061/dryad.v9s4mw6r5 (Lovas‐Kiss et al., 2020).
