# Supplementary material for: Seed mass, hardness, and phylogeny explain the potential for endozoochory by granivorous waterbirds
Source: Ecol Evol. 2020 Jan 15;10(3):1413–24. doi: 10.1002/ece3.5997 (PMC7029096; doi:10.1002/ece3.5997)
Supplement: Supplementary file 1 [file ECE3-10-1413-s001.docx]

**Supplementary material**

**Table S1.** Seed traits for each plant species fed to mallards

| Plant species | Plant family | Wet load (kg) | Seed mass (g) | Seed volume (mm^3^) | Water permeability | Dry seed shape |
| --- | --- | --- | --- | --- | --- | --- |
| *Allium angulosum* | Amaryllidaceae | 2.391 | 1.271 | 2.328 | 1.186 | 0.045 |
| *Angelica palustris* | Apiaceae | 1.669 | 1.746 | 5.978 | 1.564 | 0.12 |
| *Astragalus contortuplicatus* | Fabaceae | 1.695 | 0.331 | 0.417 | 1.1 | 0.05 |
| *Bolboschoenus planiculmis* | Cyperaceae | 6.615 | 2.749 | 6.192 | 1.155 | 0.069 |
| *Cirsium brachycephalum* | Asteraceae | 0.471 | 0.735 | 1.703 | 1.234 | 0.087 |
| *Cuscuta lupuliformis* | Convolvulaceae | 13.92 | 5.667 | 7.69 | 1.07 | 0.025 |
| *Cyperus flavescens* | Cyperaceae | 0.168 | 0.079 | 0.016 | 1.178 | 0.043 |
| *Echinochloa crus-galli* | Poaceae | 1.264 | 1.886 | 3.982 | 1.19 | 0.069 |
| *Elatine hungarica* | Elatinaceae | 0.052 | 0.008 | 0.031 | 1.116 | 0.059 |
| *Elatine hydropiper* | Elatinaceae | 0.05 | 0.017 | 0.029 | 1.057 | 0.059 |
| *Glycyrrhiza echinata* | Fabaceae | 11.713 | 11.737 | 18.914 | 1.058 | 0.034 |
| *Lychnis coronaria* | Caryophyllaceae | 0.731 | 0.421 | 0.524 | 1.185 | 0.019 |
| *Sparganium erectum* | Cyperaceae | 13.235 | 39.831 | 202.339 | 1.214 | 0.048 |

**Table S2.** Pairwise Pearson correlation tests between seed traits. Df is 11 in each case. Values represent correlation coefficients (and their p-values). Traits were log-transformed prior to the tests.

|  | **Seed volume** | **Thousand seed mass** | **Dry load** | **Wet load** | **Water permeability** | **Dry shape** | **Wet shape** |
| --- | --- | --- | --- | --- | --- | --- | --- |
| **Seed volume** | **1** (<0.0001) | **0.96** (<0.0001) | **0.91** (<0.0001) | **0.9** (<0.0001) | **0.24** (0.4263) | **0.09** (0.7804) | **0.06** (0.8573) |
| **Thousand seed mass** |  | **1** (<0.0001) | **0.94** (<0.0001) | **0.95** (<0.0001) | **0.19** (0.5321) | **0.01** (0.9635) | **0.05** (0.8694) |
| **Dry load** |  |  | **1** (<0.0001) | **0.98** (<0.0001) | **0.16** (0.6002) | **0.13** (0.6715) | **0.23** (0.4516) |
| **Wet load** |  |  |  | **1** (<0.0001) | **0.04** (0.886) | **0.08** (0.7989) | **0.19** (0.5238) |
| **Water permeability** |  |  |  |  | **1** (<0.0001) | **0.11** (0.725) | **0.47** (0.1028) |
| **Dry shape** |  |  |  |  |  | **1** (<0.0001) | **0.36** (0.2258) |
| **Wet shape** |  |  |  |  |  |  | **1** (<0.0001) |
